# Prior Use of Antiplatelet Therapy and Outcomes after Endovascular Therapy in Acute Ischemic Stroke Due to Large Vessel Occlusion: A Single-Center Experience

**DOI:** 10.3390/jcm7120518

**Published:** 2018-12-05

**Authors:** Giovanni Merlino, Massimo Sponza, Gian Luigi Gigli, Simone Lorenzut, Alessandro Vit, Vladimir Gavrilovic, Andrea Pellegrin, Daniela Cargnelutti, Mariarosaria Valente

**Affiliations:** 1 Stroke Unit, Department of Neurosciences, Udine University Hospital, 33100 Udine, Italy; simone.lorenzut@asuiud.sanita.fvg.it (S.L.); daniela.cernelutti@asuiud.sanita.fvg.it (D.C.); 2 Division of Vascular and Interventional Radiology, Udine University Hospital, 33100 Udine, Italy; massimo.sponza@asuiud.sanita.fvg.it (M.S.); alessandro.vit@asuiud.sanita.fvg.it (A.V.); vladimir.gavrilovic@asuiud.sanita.fvg.it (V.G.); andrea.pellegrin@asuiud.sanita.fvg.it (A.P.); 3 Neurology Unit, Department of Medical, University of Udine Medical School, 33100 Udine, Italy; gigli@uniud.it (G.L.G.); valente@uniud.it (M.V.)

**Keywords:** antiplatelet therapy, thrombectomy, bridging therapy, symptomatic intracranial hemorrhage, outcome, mortality

## Abstract

Endovascular therapy (EVT) represents the gold standard treatment in patients affected by acute ischemic stroke (AIS) due to large vessel occlusion (LVO). Prior antiplatelet (APT) therapy might increase the risk of bleeding and modify the clinical outcome of AIS patients treated with EVT. Thus, we compared effectiveness and safety of EVT in Caucasian patients with and without previous use of APT agents. We recruited consecutive patients admitted to Udine University Hospital with AIS undergoing EVT from January 2015 to December 2017. The following outcomes were documented: successful recanalization, 3-month favorable outcome, symptomatic intracranial hemorrhage (sICH), parenchymal hematoma (PH), and 3-month mortality. The study population included 100 patients (mean age 70.1 ± 11.2 years, 58% males). At time of admission, 34 patients were taking APT agents. Patients on APT pretreatment were older, had more vascular risk factors, and showed higher levels of serum creatinine than APT naïve patients. Moreover, prior APT therapy was associated with a higher rate of pre-stroke disability and a more severe stroke at admission. Patients pretreated with APT had higher rates of successful recanalization (91.2% vs. 74.2%, *p* = 0.04). Prevalence of 3-month unfavorable outcome and 3-month mortality was significantly higher in APT-pretreated patients than in those without APT pretreatment. However, these associations were not confirmed on multivariable analyses. Prevalence of sICH and PH did not differ in the two groups. APT pretreatment is associated to successful recanalization rate, without increasing the risk of intracranial bleeding in patients with LVO undergoing EVT.

## 1. Introduction

Endovascular therapy (EVT) represents the gold standard treatment in patients affected by acute ischemic stroke (AIS) due to large vessel occlusion (LVO) [1]. Randomized controlled trials showed that EVT in addition to best medical treatment improved clinical outcome and did not increase the rate of symptomatic intracranial hemorrhage (sICH) in this population [2,3,4,5,6]. However, prior antiplatelet (APT) therapy might increase the risk of bleeding and modify the clinical outcome of AIS patients treated with EVT.

Among patients undergoing intravenous thrombolysis (IVT), those receiving APT therapy before the stroke have a better outcome but a higher incidence of sICH than those who were not receiving APT drugs [7,8]. Little is known regarding safety and outcome of AIS patients treated with EVT on APT therapy. In fact, previous studies focused their interest on the risk of sICH after EVT while on anticoagulants [9]. Broeg-Morvay et al. compared bleeding complications and outcome between APT-naïve patients and those on APT therapy after bridging therapy (IVT + EVT). The authors reported that were no differences in bleeding complications and mortality, but outcome at 3 months was more favorable in APT-naïve patients. Since a large part of the sample was treated with acute administration of aspirin during EVT in order to prevent acute re-occlusion of extra- and/or intracranial stents, the results of Broeg-Morvay et al. cannot be generalized to AIS patients undergoing EVT on long-term APT therapy [10]. A recent Japanese study reported that prior APT therapy was an independent predictor of sICH after EVT in patients with LVO [11]. To date, only Pandhi et al. investigated the relationship of prior APT use with efficacy and safety outcomes in patients with LVO treated with EVT. In this study, APT pretreatment did not increase the risk of sICH and improved the odds of successful recanalization [12]. Although these results are very interesting, more than half of patients were African-Americans. Thus, we decided to compare effectiveness and safety of EVT in Caucasian patients with and without previous use of APT agents.

## 2. Methods

### 2.1. Patients

This study was based on a retrospective analysis of a prospectively collected database of consecutive patients admitted to the Udine University Hospital with AIS undergoing EVT from January 2015 to December 2017. Eligibility criteria for EVT were the following: (1) presence of LVO in the anterior or posterior circulation as revealed by CT angiography (CTA); (2) symptoms onset within 6 hours for LVO in the anterior circulation and within 8 hours for LVO in the posterior one; and (3) Alberta Stroke Program Early CT Score (ASPECTS) >6 on direct CT scan [13]. Patients showing symptoms onset within 4.5 hours received IVT in accordance with international guidelines [14,15].

Since anticoagulation may affect our outcomes, AIS patients treated with EVT while on therapeutic anticoagulation were excluded from the study (*n* = 23).

Informed consent was obtained from the patients or their representatives.

### 2.2. Data Collection

Baseline characteristics such as demographic data, vascular risk factor, laboratory findings, blood pressure, and pharmacological treatment at admission were collected. APT use prior to hospitalization was self-reported by patients or families during medical history collection. The TOAST classification was used to determine AIS subtypes based on their etiology [16]. Stroke severity was determined with the National Institute of Health Stroke Scale (NIHSS) score at admission. Functional outcome was assessed by means of the modified Rankin Scale (mRS) at admission based on pre-stroke disability and 3 months after stroke. The mRS score after discharge was recorded at the patients’ routine clinical visit or through telephone interview with patients or their immediate caregivers. The mRS score was dichotomized into: favorable outcome (0–2) and poor outcome (3–6). Presence of intracranial hemorrhage (ICH) was detected. We collected data on: (1) sICH based on European Cooperative Acute Stroke Study (ECASS) III protocol [17]; and (2) any parenchymal hematoma (PH) based on the ECASS morphologic definitions (ECASS PH-1 or PH-2) [18]. Relevant information regarding EVT was collected; in particular, recanalization rate was assessed at the end of EVT using the Thrombolysis in Cerebral Infarction (TICI) classification [19]. Successful recanalization was defined as TICI 2b-3. Location of the occlusion was distinguished in anterior versus posterior circulation occlusion. Presence of tandem occlusion was defined as simultaneous presence of both intracranial and extracranial arterial occlusions.

### 2.3. Outcome Measures

The efficacy endpoints were: successful recanalization and 3-month favorable outcome. The safety endpoints were: sICH, PH, and 3-month mortality.

### 2.4. Statistical Analysis

The two patient groups (APT pretreatment versus no APT pretreatment) were compared regarding baseline characteristics, treatment data and outcomes. Differences between the two groups were assessed by means of the chi-square test (Fisher’s exact test) for categorical variables and Student *t* test for independent samples when continuous variables had a normal distribution. The Mann–Whitney *U* test was used when continuous variables had an abnormal distribution and for ordinal variables. Binary logistic regression modeling was used to test the association of prior APT use with outcome measures, after adjusting for the other variables with a probability value <0.1 in univariate analysis.

Since IVT performed before EVT may increase the rate of successful recanalization and ICH in AIS patients with prior use of APT therapy, we tested these hypotheses comparing APT-pretreated patients undergoing bridging therapy and DMT.

Data are displayed in tables as means and standard deviations (SD), if not otherwise specified. All probability values are two-tailed. A *p* value <0.05 was considered statistically significant. Statistical analysis was carried out using the IBM SPSS Statistics, Version 22.0 (Chicago, IL, USA).

### 2.5. Ethical Approval

All procedures performed in studies involving human participants were in accordance with the ethical standards of the institutional and with the 1964 Helsinki declaration and its later amendments or comparable ethical standards.

## 3. Results

During the study period we recruited 100 AIS patients with LVO treated with EVT (mean age 70.1 ± 11.2 years, 58% males). Eleven patients had a pre-stroke mRS of more than 2. Median NIHSS score at admission was 18 (IQR 14–22). Bridging therapy (IVT + EVT) was performed in 60 patients, while EVT without IVT pretreatment, i.e., direct mechanical thrombectomy (DMT), was performed in 40 subjects. Occlusion site was more commonly in the anterior circulation (80%). Successful recanalization was achieved in 80% of the sample. Less than half of AIS patients (32%) had a favorable outcome after 3 months. The rates of sICH and 3-month mortality was 5% and 21%, respectively.

Out of 100 patients, 66 did not use any APT therapy at time of admission. The remaining 34 patients were receiving the following APT agents: 27 took aspirin, 6 took clopidogrel, and 1 took an aspirin + clopidogrel combination. Table 1 reports baseline characteristics in patients with and without previous use of APT agents. Patients on APT pretreatment were older, had more vascular risk factors (previous stroke and hypertension), and showed higher levels of serum creatinine than APT naïve patients. Moreover, prior APT therapy was associated with a higher rate of pre-stroke disability and a more severe stroke at admission. Table 2 summarizes information on EVT in the two groups. Patients with APT pretreatment had shorter mean times from symptom onset to EVT than those APT naïve.

As reported in Table 3, patients pretreated with APT had higher rates of successful recanalization, but worse outcomes than subjects without APT pretreatment. Prevalence of sICH and PH did not differ in the two groups. Significant predictors of efficacy and safety endpoints other than APT pretreatment by univariate analyses were the following: stroke due to large artery atherosclerosis (odds ratio, OR 0.76, 95% confidence interval, 95% CI 0.67–0.86, *p* = 0.02) for successful recanalization; age (OR 0.93, 95% CI 0.88–0.97, *p* < 0.001), stroke due to undetermined etiology (OR 2.40, 95% CI 1.01–5.71, *p* = 0.04), NIHSS score at admission (OR 0.92, 95% CI 0.85–0.98, *p* = 0.01) and pre-stroke mRS 0–2 (OR 1.56, 95% CI 1.33–1.82, *p* = 0.01) for favorable outcome at 3-months; and age (OR 1.09, 95% CI 1.01–1.16, *p* = 0.02), serum glucose (OR 1.01, 95% CI 1.00–1.02, *p* = 0.04), pre-stroke mRS 0–2 (OR 0.11, 95% CI 0.03–0.41, *p* = 0.001), sICH (OR 6.11, 95% CI 1.03–58.2, *p* = 0.01) and procedure length (OR 1.01, 95% CI 1.00–1.03, *p* = 0.01) for mortality at 3-months.

Multivariable analyses for efficacy and safety outcomes are shown in Table 4. The statistical models confirmed that prior use of APT therapy was an independent predictor of successful recanalization, whereas the association of APT pretreatment with 3-month disability and 3-month mortality was not retained.

IVT performed before EVT did not affect the association between APT pretreatment and successful recanalization. In fact, the rates of TICI 2b-3 were similar (*p* = 0.9) in APT-pretreated patients receiving bridging therapy (90.9%) and DMT (91.7%). Moreover, IVT pretreatment did not increase the risk of SICH in patients on APT therapy. In fact, a single case of sICH was observed in APT-pretreated patients occurring after DMT.

## 4. Discussion

Our study demonstrated that pre-treatment with APT improved successful recanalization rate without any increase in sICH occurrence in Caucasian patients undergoing EVT for AIS due to LVO. However, in our sample these encouraging results did not lead to better functional outcomes, because of much worse pre-stroke clinical conditions observed in patients pretreated with APT.

Aspirin, the most commonly used APT drug in our cohort, alters clot structure resulting in the formation of fibrin characterized by increased fiber thickness, large pores and reduced rigidity. Perfusion of plasminogen and plasminogen activators may interfere with these altered clots with a consequent enhancement of lysis properties [20]. This in-vitro evidence was confirmed by Sanak et al. in sample of 146 consecutive AIS patients presenting with occluded middle cerebral artery and treated with IVT. The authors demonstrated that previous APT use was associated with a higher rate of early recanalization [21]. To date, only Pandhi et al. evaluated the rate of successful recanalization after EVT in patients on long-term APT treatment. Pretreatment with APT was independently associated with increased likelihood of successful recanalization, but the authors observed that the positive effect of APT therapy on recanalization was limited only to subjects treated with bridging therapy [12]. In our cohort, prior use of APT therapy increased more than 3 times the odds of successful recanalization in patients treated with EVT. However, successful recanalization rates did not differ between AIS patients undergoing bridging therapy and DMT.

Since APT therapy decreases platelet aggregation and inhibits thrombus formation, AIS patients pretreated with APT might have an increased risk for bleeding after reperfusion therapy. The largest study evaluating outcomes associated with prestroke APT therapy among AIS patients treated with IVT showed that subjects with APT pretreatment had higher risks of sICH, but better functional outcomes after tissue plasminogen activator [8]. Sogiura et al. investigated the predictors of sICH after EVT. Hyperglycemia, high baseline NIHSS score and prior APT therapy were associated with sICH. In particular, APT pretreatment increased more than 8 times the odds of sICH in patients undergoing EVT [11]. This strong association should be considered with caution, because the majority of patients were treated using obsolete reperfusion strategies, such as intra-arterial lytics and mechanical disruption, that have been associated with higher risks of sICH than using the newer generation endovascular devices [22]. Moreover, the study was performed in Asian patients that are affected by ICH more frequently than other populations [23]. Prior use of APT therapy did not increase the risk of ICH in our cohort of Caucasian patients who underwent EVT. We observed a not significant trend for sICH and PH events more common in patients without APT pretreatment than in those pretreated with APT.

Similar results were reported by Pandhi et al. in Caucasian/African-American patients [12]. Broeg-Morvay et al. analyzed bleeding complications after bridging therapy for AIS in APT-naïve patients with those with prior or acute APT therapy. The authors did not observe any association between prior APT therapy and sICH occurrence [10].

Although APT pretreatment enhanced recanalization of LVO in our sample of patients treated with EVT, we observed that subjects with prior use of APT had worse outcomes, in terms of disability and mortality, than those APT-naïve. As shown by the multivariable analyses, these differences were due to the fact that patients with APT pretreatment were older, had more severe strokes at admission, and more severe pre-stroke disabilities. Similarly, Broeg-Morvain et al. reported that outcomes were significantly worse in patients with prior APT treatment because they were older, had more comorbidities, and more severe strokes [10].

Several limitations of this study need to be acknowledged. First, this is a retrospective monocenter analysis of prospectively collected data. Second, the numerosity of our sample made impossible to perform additional subgroup analysis for patients with dual APT therapy. Third, data on infarct volume at baseline and peri-procedural blood pressure were not collected in our dataset; both factors may have an important impact on functional outcome and bleeding risk.

## 5. Conclusions

Prior use of APT therapy improves successful recanalization rate and does not increase the risk of intracranial bleeding in patients affected by AIS due to LVO and treated with EVT. Since patients pretreated with APT are likely to be older and to have more comorbidities and severe strokes, their clinical outcomes may be poorer compared with APT-naïve patients. Further studies with larger patient numbers and prospective design are needed to confirm our findings.

## Figures and Tables

**Table 1:** Baseline characteristics.

	APT Pretreatment (*n* = 34)	No APT Pretreatment (*n* = 66)	*p*
**Demographic Data and Baseline Clinical Characteristics**			
Males, *n* (%)	20 (58.8)	38 (57.6)	0.9
Age, years	73.9 ± 9.0	68.5 ± 11.8	0.02
NIHSS score at admission, median (IQR)	20 (17.5–23)	16 (11–19)	0.002
pre-stroke mRS 0–2, *n* (%)	25 (73.5)	64 (97.0)	0.001
**Vascular Risk Factors**			
Previous stroke, *n* (%)	4 (11.8)	1 (1.5)	0.04
Atrial fibrillation, *n* (%)	14 (41.2)	27 (40.9)	0.9
Hypertension, *n* (%)	30 (88.2)	37 (56.1)	0.01
Diabetes mellitus, *n* (%)	9 (26.5)	9 (13.6)	0.1
Hypercholesterolemia, *n* (%)	12 (35.3)	13 (19.7)	0.08
Current smoking, *n* (%)	4 (11.8)	6 (9.1)	0.7
**Laboratory Findings**			
Serum glucose, mg/dl	155.6 ± 55.3	137.9 ± 43.4	0.1
Creatinine, mg/dl	1.05 ± 0.2	0.90 ± 0.2	0.008
aPTT ratio	0.92 ± 0.1	0.93 ± 0.1	0.9
**Blood Pressure**			
SBP, mmHg	142.5 ± 22.9	146.9 ± 21.9	0.4
DBP, mmHg	78.6 ± 14.2	81.3 ± 13.6	0.4
**Stroke Subtypes Based on TOAST Classification**			
Large artery atherosclerosis, *n* (%)	8 (23.5)	9 (13.6)	0.2
Cardioembolism, *n* (%)	15 (44.1)	28 (42.4)	0.9
Other determined etiology, *n* (%)	0 (0)	4 (6.1)	0.3
Undetermined etiology	11 (32.4)	25 (37.9)	0.6

APT = antiplatelet; NIHSS = National Institute of Health Stroke Scale; mRS = modified Rankin Scale; aPTT = activated partial thromboplastin time; SBP = systolic blood pressure; DBP = diastolic blood pressure.

**Table 2:** Information on endovascular therapy (EVT).

	APT Pretreatment (*n* = 34)	No APT Pretreatment (*n* = 66)	*p*
Bridging therapy, *n* (%)	22 (64.7)	38 (57.6)	0.5
Occlusion in anterior circulation, *n* (%)	29 (85.3)	51 (77.3)	0.3
Presence of tandem occlusions, *n* (%)	3 (8.8)	10 (15.2)	0.5
Mean time from symptom onset to IVT, min	143.3 ± 40.0	146.3 ± 45.3	0.8
Mean time from symptom onset to EVT, min	202.8 ± 64.5	252.1 ± 91.3	0.002
Procedure length, min	88.8 ± 41.4	86.8 ± 38.9	0.8

APT = antiplatelet; IVT = intravenous thrombolysis; EVT = endovascular therapy.

**Table 3:** Efficacy and safety endpoints.

	APT Pretreatment (*n* = 34)	No APT Pretreatment (*n* = 66)	*p*
**Efficacy Endpoints**			
Successful recanalization, *n* (%)	31 (91.2)	49 (74.2)	0.04
Favorable outcome at 3-months, *n* (%)	6 (17.6)	26 (39.4)	0.02
**Safety Endpoints**			
sICH, *n* (%)	1 (2.9)	4 (6.1)	0.6
PH, *n* (%)	2 (5.9)	6 (9.1)	0.7
Mortality at 3-months, *n* (%)	11 (32.4)	10 (15.2)	0.04

APT = antiplatelet; sICH = symptomatic intracranial hemorrhage; PH = parenchymal hematoma.

**Table 4:** Multivariable analyses showing the independent predictors of successful recanalization, favorable outcome at 3 months and mortality at 3 months.

**Successful Recanalization ^a^**	OR	95% CI	*p*
APT pretreatment	3.58	1.41–13.25	0.04
**Favorable Outcome (mRS 0–2) at 3-Months ^b^**	OR	95% CI	*p*
Age (per 1 year increase)	0.93	0.89–0.94	0.009
Stroke due to undermined etiology	3.09	1.11–8.58	0.03
NIHSS at admission (per 1 point increase)	0.92	0.85–0.99	0.04
**Mortality at 3-Months ^c^**	OR	95% CI	*p*
Pre-stroke mRS 0–2	0.15	0.02–0.99	0.05
sICH	11.22	1.17–153.56	0.008
Procedure length (per 1 minute increase)	1.01	1.00–1.03	0.04

^a^ OR was adjusted for the following variable: stroke due to large artery atherosclerosis. ^b^ ORs were adjusted for the following variables: pre-stroke mRS, prior antiplatelet use. ^c^ ORs were adjusted for the following variables: age, serum glucose, prior antiplatelet use. OR = odds Ratio; CI = confidence interval; APT = antiplatelet; NIHSS = National Institute of Health Stroke Scale; mRS = modified Rankin Scale; sICH = symptomatic intracranial hemorrhage.
